# Coeliac disease hidden by cryptogenic hypertransaminasaemia in children: a case report

**DOI:** 10.11604/pamj.2022.41.27.29084

**Published:** 2022-01-11

**Authors:** Brahim El Hasbaoui, Jihane El Mahi, Rachid Abilkassem, Aomar Agadr

**Affiliations:** 1 Department of Pediatrics, Military Teaching Hospital Mohammed V, Faculty of Medicine and Pharmacy, University Mohammed V, Rabat, Morocco,; 2 Department of Pediatrics, Children´s Hospital, Faculty of Medicine and Pharmacy, University Mohammed V, Rabat, Morocco

**Keywords:** Coeliac hepatitis, hypertransaminasemia, gluten-free diet, liver abnormality, case report

## Abstract

Celiac disease is a chronic immune-mediated multisystem disorder that may affect several organs. Isolated hypertransaminasemia, with mild or nonspecific histologic changes in the liver biopsy, also known as “celiac hepatitis”, is the most frequent presentation of liver injury in celiac disease. Both, histologic changes and liver enzymes reverse to normal after treatment with a gluten-free diet in most patients. Here we report the case of a young boy presenting with asymptomatic and persistent hypertransaminasemia whose etiologic investigation led to the diagnosis of celiac disease that resolved with dietary treatment alone. This case emphasizes the need to screen Celiac disease in patients with cryptogenic hypertransaminasemia, irrespective of the existence of gastrointestinal symptoms. It also exemplifies a particular situation in which a liver biopsy is useful to establish the diagnosis of celiac hepatitis.

## Introduction

Celiac disease (CD), also known as gluten-sensitive enteropathy or celiac sprue, is defined as a permanent intolerance to ingested gluten (the storage protein components of wheat, barley, and rye). This food intolerance, whose prevalence is very high in the general population (1 in 100-150 people), is nowadays considered as a result of a complex interplay between intrinsic and extrinsic factors. In contrast to most other immune system disorders, the trigger (gliadin), a close genetic association (human leukocyte antigen [HLA]-DQ2 or HLA-DQ8), and the specific autoantigen (tissue transglutaminase-tTG) is well known.

Although CD is defined by the small intestine injury and resulting malabsorption, more recently it has been recognized to be a multisystem disorder that may affect other organs, such as the nervous system, bones, skin, heart, and, the liver. Several hepatic disorders have been described in association with celiac disease. Isolated hypertransaminasemia with nonspecific histologic changes in a liver biopsy is the commonest hepatic presentation of celiac disease. A gluten-free diet normalizes liver enzymes and histologic changes in most patients. Here we present a case of coeliac hepatitis in a 7-year-old boy who had been referred due to persistent hypertransaminasemia.

## Patient and observation

**Patient information:** a 7-year-old male was referred for evaluation because of an unclear and enduring elevation of liver enzymes. The boy was born at full term of non-consanguineous parentage with no antenatal or perinatal complications, He was exclusively breast-fed, food diversification was started at 6 months old, his weight, length, and psychomotor development were within the normal range, the child was described as a good eater, was on a normal diet, and was thriving appropriately. Furthermore, the boy presented abdominal distension with recurrent episodes of mild diarrhea since the last 6 months. He was not taking any medications, including nonprescription ones. There was no history of jaundice or bleeding from any site. Family history was negative for neoplastic and autoimmune disorders.

**Clinical results:** there were no signs of liver disease either at the physical examination or at the Abdominal ultrasound.

**Diagnostic procedure:** the initial laboratory study evidenced AST 120 U/L (upper limit of reference, 31 U/L) and ALT 134 U/L (upper limit of reference, 34 U/L), with normal alkaline phosphatase, gamma-glutamyl transferase, bilirubin, and normal hemogram. A complete screen for the etiology of abnormal liver tests was performed including Serologic markers for viral hepatitis were negative, Transferrin saturation, ferritin, ceruloplasmin, alpha1-antitrypsin, and thyroid function tests were normal. The serum protein electrophoresis and immunoglobulin study disclosed an elevation of serum immunoglobulin (Ig) G concentrations (19.7g/L; normal 7-16g/L) and low serum IgA (0.24g/L; normal 0.7-4g/L).

**Figure 1:**
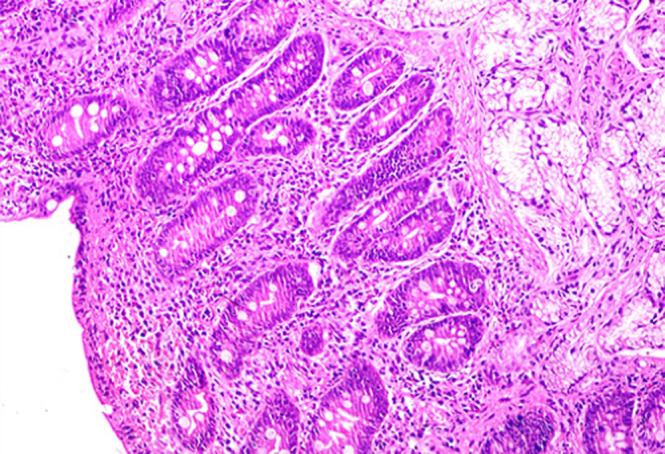
duodenal biopsy showing severe villous abnormality, increased intraepithelial lymphocytes and crypt hyperplasia

**Figure 2:**
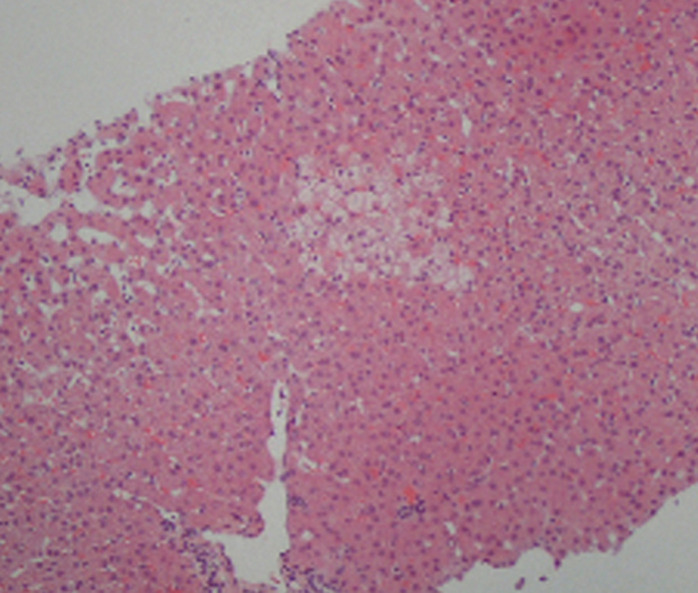
liver biopsy showing nonspecific changes with ballooned hepatocytes

The autoantibody profile was characterized by negatives antinuclear, anti-double-strand DNA, and anti-smooth muscle antibodies, plus a positivity to IgG anti-transglutaminase (528 U/mL; positive, >10 U/mL) and anti-gliadin antibodies (600 U/mL; positive > 10 U/mL); anti-endomysial antibodies were negative. The patient underwent an upper gastrointestinal endoscopy, which showed a slight loss of folds in the second portion of the duodenum. Multiple biopsies were obtained in this location, revealing complete villous atrophy, crypt lengthening, and markedly increased number of intraepithelial lymphocytes (Figure 1), histopathological findings typical of celiac disease (with a destructive pattern, 3c type according to the Marsh-Oberhuber classification). It was decided to perform a liver biopsy that revealed minimal macrovesicular steatosis and hepatocellular reactive changes, with no evidence of inter-face hepatitis (Figure 2), all nonspecific findings, not consistent with Autoimmune hepatitis.

**Therapeutic intervention and follow-up:** the child received dietary counseling and started on a strict gluten-free diet alone. After 6 months the laboratory reassessment evidenced a complete normalization of aminotransferases (AST 25U/L, ALT 22 U/L) and decreasing IgG anti-transglutaminase levels (42 U/mL). The trends of hematological and biochemical parameters before and after gluten-free diet are summarized in Table 1.

**Table 1:** the trends of the hematological and biochemical parameters before and after gluten-free diet

Biological parameters	Before gluten-free diet	After gluten-free diet
Hemoglobin	7g/dl	11g/dl
Ferritin	6ng/ml	40ng/ml
Alanine aminotransferase	172 UI/L	35UI/L
Aspartate aminotransferase	50UI/L	30UI/L

**Patient perspective:** during hospital stay and at discharge, the patient´s mother was delighted with the care.

**Informed consent:** the patient´s mother was informed about the report, why this case was peculiar and the authors' interest in publishing it. She gave informed consent to allow the authors to use her case for this case report.

**Patient's consent:** informed consent was obtained from the patient's parent for us to use the case.

## Discussion

Celiac disease is a chronic intestinal inflammatory disease that manifests in genetically susceptible individuals when exposed to dietary gluten. Liver abnormalities are common extraintestinal manifestations in patients with Celiac disease and range from mild hepatic injury to severe liver disease [1]. The prevalence of hypertransaminasemia in patients with Celiac disease ranges between 15% and 61%, with the highest prevalence observed in children. Conversely, the prevalence of Celiac disease in patients with unexplained hypertransaminasemia is 10%, a finding that justifies screening for Celiac disease in all patients with abnormal liver biochemical test results [2]. An association between Celiac disease and cryptogenic liver damage was first reported in 1977, finding out that 40% of adults with incipient Celiac disease had increased serum concentrations of transaminases. One year later, Farre *et al*. reported elevation of serum aminotransferases in about one-third of pediatric patients with Celiac disease [3].

The mechanism(s) underlying liver injury in Celiac disease are poorly understood. Serum aminotransferase elevations will normalize with the removal of gluten from the diet, this suggests a causal relationship between gluten intake/intestinal damage and liver injury. Intestinal permeability is increased in Celiac disease; this is related either to the inflammation of the intestine or the induction of the secretion of zonulin, a regulator of tight junctions. Patients with Celiac disease and hypertransaminasemia show a significant increase in intestinal permeability in comparison with those with normal liver tests. The increased intestinal permeability seen in the context of Celiac disease may facilitate the entry of toxins, antigens, and inflammatory substances (cytokines and/or autoantibodies) to the portal circulation, and these mediators may have a role in the liver involvement seen in patients with Celiac disease. Autoantibodies directed against the so-called celiac antigen (tissue transglutaminase) are present in the liver and other extraintestinal tissues in Celiac disease, raising the possibility of a pathogenic role for the humoral-mediated immune responses in the liver injury observed in Celiac disease [4].

The clinical picture is polymorphic, is characterized by various intestinal (diarrhea, constipation, bloating, abdominal pain) and extraintestinal symptoms/signs (fatigue, anemia, aphthous stomatitis, osteopenia/osteoporosis, recurrent miscarriages, and hypertransaminasemia) or by the absence of any symptom in most of the cases. Diagnostic criteria have been recently revised, but the two most relevant elements remain serology and small intestinal biopsy. Anti-transglutaminase antibodies and endomysial antibodies of the IgA class show the highest diagnostic accuracy for Celiac disease, displaying a very high sensitivity (up to 98%) and specificity (from 90 to 99%) [5].

Small bowel biopsy showing different grades of mucosal damage ranging from flat mucosa to mild intestinal lesions, characteristically inducing villous atrophy and crypt hyperplasia is still regarded as the diagnostic gold standard in adults, whereas in children and adolescents, the European Society of Paediatric Gastroenterology, Hepatology and Nutrition has recently proposed that Celiac disease diagnosis can be accepted without the need of duodenal biopsy in selected cases showing anti-transglutaminase antibodies at a high titer (>ten-times upper normal limit), confirmed by endomysial antibodies and HLA-DQ2 and/or -DQ8 positivity [6]. Mild to moderate elevated serum levels (less than 5 times the upper limit of normal) of aspartate aminotransferase and/or alanine aminotransferase are the most common and often only laboratory manifestation of liver injury in patients with Celiac disease. While Ultrasonographic findings vary from a normal to coarse/heterogeneous liver echotexture, depending on the degree of liver injury [7, 8].

Histologic Findings in patients with Celiac disease and liver injury for whom a liver biopsy has been performed, histological changes are frequent (66%) but generally mild and nonspecific [9]. The abnormalities include commonly peri-portal inflammation, bile duct obstruction, increased number of Kupffer cells, mononuclear infiltration in the parenchyma, steatosis, and mild fibrosis. Extensive fibrosis and cirrhosis have also been reported [10]. Liver biopsy may also be useful in coexisting specific hepatic disorder or when there is a lack of response to diet. A gluten-free diet leads to normalization of serum transaminases and liver histological changes in 75% to 95% of patients with Celiac disease, usually within a year of good adherence. In those patients with persistent elevations despite good compliance to gluten exclusion, an alternative etiology should be investigated.

## Conclusion

The present case illustrates the association between Celiac disease and abnormal liver tests. Our patient was a young boy presenting with enduring hypertransaminasemia whose etiologic investigation led to the diagnosis of celiac disease that resolved with dietary treatment alone. This case emphasizes the need to screen Celiac disease in patients with cryptogenic hypertransaminasemia, irrespective of the existence of gastrointestinal symptoms. It also exemplifies a particular situation in which a liver biopsy is useful to establish the diagnosis of celiac hepatitis.
